# The effects of pomegranate consumption on obesity indices in adults: A systematic review and meta‐analysis

**DOI:** 10.1002/fsn3.3739

**Published:** 2023-11-23

**Authors:** Hossein Bahari, Sanaz Pourreza, Kian Goudarzi, Seyedeh Nooshan Mirmohammadali, Omid Asbaghi, Kosar Sadat Hosseini Kolbadi, Moslem Naderian, Ali Hosseini

**Affiliations:** ^1^ Department of Nutrition, Faculty of Medicine Mashhad University of Medical Sciences Mashhad Iran; ^2^ Student Research Committee Mashhad University of Medical Sciences Mashhad Iran; ^3^ Department of Community Nutrition, School of Nutritional Sciences and Dietetics Tehran University of Medical Sciences Tehran Iran; ^4^ Faculty of Medicine Shahid Beheshti University of Medical Science Tehran Iran; ^5^ Department of Food, Nutrition, Dietetics and Health Kansas State University Manhattan Kansas USA; ^6^ Cancer Research Center Shahid Beheshti University of Medical Sciences Tehran Iran; ^7^ Student Research Committee Shahid Beheshti University of Medical Sciences Tehran Iran; ^8^ Faculty of Medicine Iran University of Medical Science Tehran Iran; ^9^ Department of Pharmacognosy, School of Pharmacy Shiraz University of Medical Sciences Shiraz Iran; ^10^ Medicinal Plants Research Center Yasuj University of Medical Sciences Yasuj Iran

**Keywords:** body composition, meta‐analysis, obesity, pomegranate

## Abstract

Evidence supports the potential application of polyphenols as agents against obesity. Pomegranate is one of the fruits that possess a high content of polyphenols. This systematic review and meta‐analysis of randomized controlled trials (RCTs) sought to evaluate the effects of pomegranate consumption on obesity indices, including body mass index (BMI), body weight, waist circumference (WC), fat mass (FM), body fat percentage (BFP), and fat‐free mass (FFM) in adults. Relevant RCTs were obtained by searching databases, including PubMed, SCOPUS, and Web of Science, up to May 2023. Heterogeneity tests of the included trials were performed using the *I* ^2^ statistic. Random effects models were assessed based on the heterogeneity tests, and pooled data were determined as the weighted mean difference with a 95% confidence interval. Pooled analysis of 28 trials revealed that pomegranate consumption led to a significant reduction in body weight (WMD: −1.97, 95% CI: −2.91, −1.03, *p* < .05), and a significant decrease in BMI (WMD: −0.48, 95% CI: −0.76, −0.20, *p* < .05) in comparison with the control group. However, there were no significant effects on WC, FM, FFM, and BFP in comparison with the control group. Pomegranate consumption may yield a beneficial effect on body weight and BMI in adults. However, there were no significant effects on WC, FM, FFM, and BFP, by pomegranate consumption. Also, pomegranate consumption can reduce body weight, BMI, WC, and BFP in obese adults. Long‐term trials with different doses of pomegranate are needed.

## Graphical abstract

In this paper, we report on the effect of pomegranate on BMI, body weight, waist circumference, fat mass, fat‐free mass, and body fat percentage. We performed a thorough systematic review and meta‐analysis of randomized controlled trials, exactly according to the Cochrane Handbook for Systematic Reviews of Interventions. This is the systematic review pooling the data of clinical trials that provides evidence for the significant improvement in BMI and body weight following pomegranate consumption.

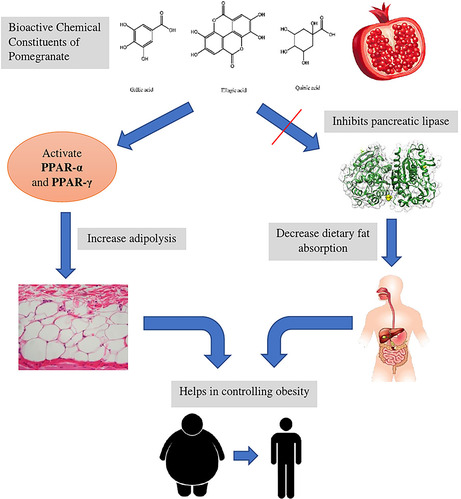

## 1 INTRODUCTION

The excessive or abnormal buildup of fat or adipose tissue in the body, which has the potential to harm health, is called obesity (Saalbach & Anderegg, 2019). The etiology of obesity is multiple, making it a complex condition. After smoking, it is the second most frequent avertable cause of mortality. Treatment for obesity should be multifaceted and may last a lifetime (Kozlov, 2019). A 5% to 10% weight loss can greatly improve a person's health, quality of life, and financial burden both personally and nationally (Gowd et al., 2019). Costs associated with obesity in healthcare surpass $700 billion annually. The financial cost alone in the United States is thought to be around $100 billion a year (Holly et al., 2019). In 2019, the United States incurred substantial annual medical care costs related to obesity, amounting to nearly $173 billion. Additionally, the national productivity costs due to obesity‐related absenteeism ranged between $3.38 billion (equivalent to $79 per person with obesity) and $6.38 billion (equivalent to $132 per person with obesity; Prevention, 2022). For the past 50 years, the epidemic of obesity has gotten worse. Almost 500 million people globally are obese, with obesity rates rising at an astounding rate (Panuganti et al., 2022). Obesity is intricately linked to a range of health conditions, either directly causing or aggravating their development. These conditions include diabetes, insulin resistance due to excess fat, and also cardiovascular disease which may cause stroke (Bahari, Taheri, et al., 2023; Bonora et al., 2007). Obesity constitutes a notable risk factor for various major cancers, such as post‐menopausal breast, colorectal, endometrial, kidney, esophageal, pancreatic, liver, and gallbladder cancer. Having excess body fat leads to an approximately 17% elevation in the risk of cancer‐specific mortality (Pati et al., 2023). The cause of obesity is an imbalance between daily energy intake and expenditure that leads to excessive weight gain. Many genetic, socioeconomic, cultural, and environmental variables contribute to obesity, which is a complex disease (Apovian, 2016). Many genes have been linked to adiposity and weight growth, proving that obesity is a highly heritable condition. The availability and consumption of excessive amounts of carbohydrates, high‐sugar foods, unhealthy dietary pattern which lacks fruit and vegetables as well as sedentary behavior, are some additional causes of obesity (Wiechert & Holzapfel, 2021). Therefore, improving dietary intake and active lifestyle could decrease body weight and prevent obesity.

Dietary instructions must be followed in order for behavioral weight loss treatment (BWL) and long‐term weight reduction maintenance to be successful (Hill et al., 2012). The fundamental factors in combating obesity involve achieving a negative energy balance by reducing calorie intake and expending calories through physical exercise. Equally important are meal portion control, a well‐balanced distribution of macronutrients, and the reduction of processed foods and added sugars. Simultaneously, increasing the consumption of nutritious foods, such as fruits and vegetables, becomes crucial as they supply essential nutrients for the body. Several fruit and vegetable‐rich diets, such as the Mediterranean diet, have been linked to weight loss since it consists of fish, monounsaturated fats from olive oil, fruits, vegetables, whole grains, legumes/nuts, and moderate alcohol consumption (Schwarzfuchs et al., 2012). The Mediterranean diet can serve as an effective approach for weight reduction, particularly when combined with energy restriction, regular physical activity, and maintained for more than 6 months. Fruits including pomegranate, apple, berries, and pear, as well as vegetables like kale, and broccoli could help weight loss (Dreher & Ford, 2020).

Pomegranate (*Punica granatum* L.) is a shrub grown in Iran, India, the Mediterranean countries, Malaysia, tropical Africa, and, to a lesser extent, the United States (Ercisli et al., 2011). The arils are deep red or purple in color due to a high polyphenol content, primarily anthocyanins (Viuda‐Martos et al., 2010). Pomegranate is a powerful antioxidant with stronger antioxidant activity than vitamins E, A, and C due to its high polyphenol, flavonoid, anthocyanin content which has anti‐obesity, anticancer, anti‐inflammatory effects by inhibiting LDL oxidative damage by reducing free radicals (Bahari, Rafiei, et al., 2023; Vučić et al., 2019). Pomegranate juice has the highest antioxidant potential of any polyphenol‐rich beverage or fruit juice ingested, including green tea, red wine, and orange, grapefruit, grape, or cranberry juice (Seeram et al., 2008). From ancient times, extracts from this plant have been used to treat a variety of illnesses including obesity, diabetes, metabolic diseases, parasitic infections, ulcers, diarrhea, dysentery, bleeding, microbiological infections, and respiratory disorders (Wong et al., 2021). Its fruit, juice, extract, leaf, and peel have been utilized for generations because of its anti‐inflammatory, antioxidant, and anticancer characteristics, as well as its neuroprotection and cardiovascular protection (Bahari, Rezaiian, et al., 2023; Barati Boldaji et al., 2020; Khajebishak et al., 2019). *P. granatum* use is also linked to anti‐diabetic benefits and modifies other parts of the metabolic syndrome diagnostic components (MetS; Sohrab et al., 2019).

Polyphenols have gotten a lot of interest as potential supplemental treatments for obesity (Meydani & Hasan, 2010). The appetite‐suppressing effects of natural polyphenolic compounds have been attributed to the interaction of various mechanisms, including slowing down the secretion of appetite‐stimulating hormones, inactivation of appetite sensors, modulation of melanin‐concentration hormone receptors, inhibition of ghrelin secretion, increase of adiponectin levels, reduction of glucagon‐like peptide 1, increase of serotonin, and modulation of adipohormones (Lai et al., 2012). Pomegranate juice and pomegranate extract consumption have also been shown in animal studies to reduce food consumption and body weight (Lei et al., 2007). Catalpic acid is a conjugated linolenic acid that is mostly present in pomegranate seeds. Hontecillas et al. (2009) demonstrated that 78 days of feeding mice 1 g of catalpic acid per 100 g of a high‐fat diet led to a decrease in the buildup of abdominal white adipose tissue, as well as improvements in fasting glucose and insulin concentrations compared to control mice. A human study by González‐Ortiz et al. (2011) found that consuming 120 mL of pomegranate juice daily for 1 month dramatically reduced fat mass. Given the varying results from different randomized clinical trials on the effect of pomegranate on body weight in adults, our goal was to conduct a systematic review and meta‐analysis of randomized controlled trials. By pooling previous findings, we aimed to arrive at a more solid and conclusive result.

## 2 MATERIALS AND METHODS

### 2.1 Search strategy and study selection

To conduct this meta‐analysis, we utilized the Preferred Reporting Items for Systematic Reviews and Meta‐Analyzes (PRISMA; Moher et al., 2009), and a comprehensive scan of the literature up to May 2023 in PubMed, Scopus, and ISI Web of Science was performed in order to spot suitable articles without restraining the language or timeline. We screened databases using these specific search terms in titles and abstracts; (pomegranate OR “punica granatum”) AND (“weight” OR “body mass index” OR “bmi” OR “waist circumference” OR “wc” OR “percentage of body fat” OR “body fat percentage” OR “fat free mass” OR “lean body mass” OR “ffm” OR “lbm” OR “fat mass” OR “fm”). The studies that were included were screened by The Endnote program.

### 2.2 Eligibility criteria

All included articles met the following criteria: (1) randomized controlled trials (RCTs) assessing the impacts of pomegranate intake on body composition in adults as an outcome (body weight, BMI, FM, FFM, BFP, WC) with a control group, (2) researches conducted on adults (≥18 years) that obtained pomegranate as an intervention, (3) experiments with at least 3 weeks of intervention time length, (4) studies with parallel or crossover structure, (5) studies with a report of the consequence at the start and the end of the intervention.

### 2.3 Exclusion criteria

All included articles with these characteristics were excluded after the full‐text analysis: (1) animal, review, ecological, and observational studies, (2) studies carried out on individuals younger than 18 years, and (3) studies with a lack of randomization, placebo or control groups.

### 2.4 Data extraction

The evaluation of studies based on titles and abstracts was done to determine eligibility. Afterward, the potential studies were examined according to their full text to decide whether they could be included in this meta‐analysis. At last, this particular information was extracted: the name of the first author, the publication year, the study location and design, the number of subjects in each group, and the details of the individuals such as mean age, gender, and body mass index, the dose of pomegranate used for the intervention, the period of the intervention, the mean change and the standard deviation of the indexes during the study for both the intervention and control groups. By determining various data for an exact study at different time points, merely the most current was regarded.

### 2.5 Quality assessment

Two distinct research experts assessed the quality level of the eligible studies utilizing the Cochran scoring methodology (Higgins & Green, 2010). This comprises seven criteria to gauge the risk of bias, which are as follows: random sequence generation, allocation concealment, blinding of subjects and staff, blinding of outcome assessment, incomplete outcome data, selective reporting, and other biases. As a result, terms such as “Low,” “High,” or “Unclear” were applied to assess each sector. Furthermore, any misleading information in the studies was clarified by corresponding authors.

### 2.6 Data synthesis and statistical analysis

In this meta‐analysis, we pooled all results of the studies to find out the overall effect sizes, weighted mean differences (WMD), and the SD of measures from both intervention and control groups were extracted, using the random‐effects model according to DerSimonian and Laird method (DerSimonian & Laird, 1986). The included studies used different types, doses, and duration of intervention and had different location of study, and the health status of participants. So, Due to this heterogeneity, we used a random‐effects model. Furthermore, without mean changes reporting, it was calculated by using this formula: mean change = final values − baseline values, and SD changes were calculated by the following formula (Borenstein et al., 2021):

$$\begin{array}{c} \mathrm{SD}\;\text{change}\\ =\sqrt{[{\left(\mathrm{SD}\;\text{baseline}\right)}^{2}+{\left(\mathrm{SD}\;\text{final}\right)}^{2}-\left(2R\times \mathrm{SD}\;\text{baseline}\times \mathrm{SD}\;\text{final]}\right)} \end{array}$$

We also converted standard errors (SEs), 95% confidence intervals (CIs), and interquartile ranges (IQRs) to SDs using the method of Hozo et al. (2005). The random‐effects model that accounted for between‐study variations was used to determine the overall effect size. In addition, the Between‐study heterogeneity was examined by Cochran's *Q* test and was measured using the *I*‐squared statistic (*I* ^2^; Higgins et al., 2003). *I* ^2^ > 40% or *p*‐value < .05 was deemed as high between‐study heterogeneity. To detect potential sources of heterogeneity (Higgins & Thompson, 2002), subgroup analyses were performed according to pre‐planned criteria, including study duration (<8 and ≥8 weeks), baseline levels of body composition indexes, and intervention doses (mg/day and mL/day). A sensitivity analysis was conducted to determine the influence of each specific study on the overall estimation. In sensitivity analysis, trials were excluded one by one, in order to see if one specific study changes the overall effect size (Tobias, 1999). The possibility of publication bias was checked by Egger's regression test and the visually inspected funnel plot test (Egger et al., 1997). Statistical analysis was executed using STATA, version 11.2 (Stata Corp). In all analyses, the *p*‐values < .05 were considered statistically significant. The quality of evidence across RCTs was rated using the Grading of Recommendations Assessment, Development, and Evaluation (GRADE) Working Group guidelines (Guyatt, 2009; Guyatt et al., 2008). According to the corresponding evaluation criteria, the quality of evidence was classified into four categories: high, moderate, low, and very low.

## 3 RESULTS

### 3.1 Study selection

**FIGURE 1:**
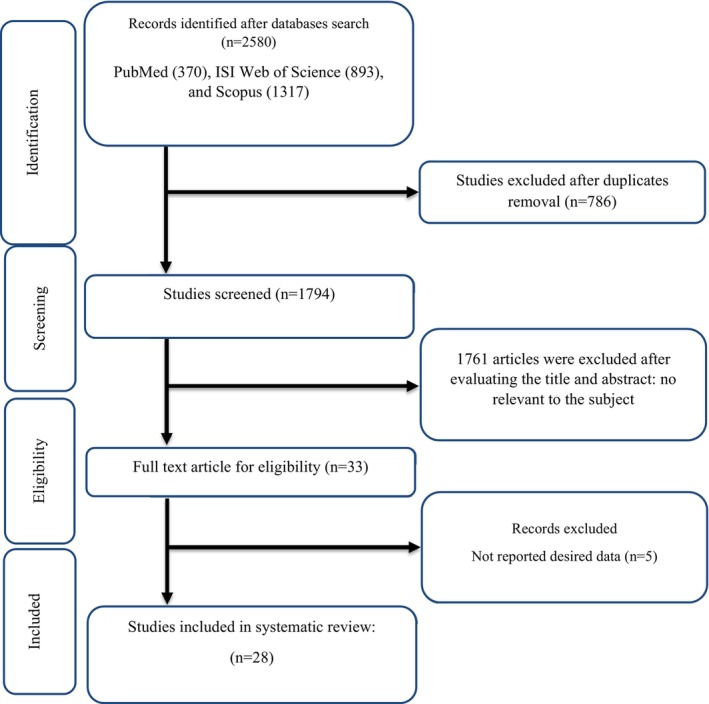
Flow chart of study selection for inclusion trials in the systematic review.

Following Figure 1, at the first step, an exhaustive search was conducted in international online datasets and 2580 studies were found. As a result, 786 duplicate trials, and 1761 studies due to irrelevancy studies were removed according to the evaluation of the titles and abstracts. Moreover, due to the lack of important data reporting following the full‐text assessment of the trials, five studies were excluded. Finally, according to the inclusion criteria, this meta‐analysis was conducted by including 28 trials.

### 3.2 Study characteristic

Ultimately this meta‐analysis review was executed by qualifying 28 articles, with 1124 participants (568 cases and 556 controls). All included studies were published between 2005 and 2022. Moreover, the intervention duration varied from 2 weeks (Kojadinovic et al., 2021; Manthou et al., 2017) to 24 (Wu et al., 2015) weeks, and the sample size differed from 10 (Manthou et al., 2017) to 77 (Park et al., 2014) participants. The design of 26 trials was parallel RCT (Abedini et al., 2021; Akbarpour et al., 2021; Al‐Dujaili et al., 2022; Babaeian et al., 2013; Esmaeilinezhad et al., 2019; Faghihimani et al., 2016; González‐Ortiz et al., 2011; Goodarzi et al., 2021; Grabež et al., 2020; Hosseini et al., 2016; Irani et al., 2020; Kojadinovic et al., 2017, 2021; Lynn et al., 2012; Manthou et al., 2017; Mirmiran et al., 2010; Nemati et al., 2022; Park et al., 2014; Rouzbehan et al., 2021; Sohrab et al., 2014, 2019; Stockton et al., 2017; Sumner et al., 2005; Wu et al., 2015; Yarmohammadi & Mahjoub, 2017; Zarezadeh et al., 2019), and the two (Barati Boldaji et al., 2020; Tsang et al., 2012) were performed in a crossover design. Included studies possessed different subjects including patients with coronary heart disease (Sumner et al., 2005), hyperlipidemic individuals (Mirmiran et al., 2010), Obese participants (González‐Ortiz et al., 2011), individuals who were at high CVD risk (Tsang et al., 2012), healthy young and middle‐aged men and women (Lynn et al., 2012), type 2 diabetic individuals (Babaeian et al., 2013; Faghihimani et al., 2016; Grabež et al., 2020; Nemati et al., 2022; Sohrab et al., 2014, 2019; Yarmohammadi & Mahjoub, 2017), overweight participants (Park et al., 2014), hemodialysis patients (Barati Boldaji et al., 2020; Wu et al., 2015), overweight and obese individuals (Hosseini et al., 2016; Zarezadeh et al., 2019), healthy participants (Al‐Dujaili et al., 2022; Manthou et al., 2017; Stockton et al., 2017), metabolic syndrome patients (Kojadinovic et al., 2017), individuals with PCOS (Abedini et al., 2021; Esmaeilinezhad et al., 2019), inactive students (Irani et al., 2020), patients with NAFLD (Goodarzi et al., 2021), overweight individuals with dyslipidemia (Kojadinovic et al., 2021), and patients with breast cancer (Akbarpour et al., 2021; Rouzbehan et al., 2021). The main countries which included trials were mainly executed in, were the USA (Sumner et al., 2005; Wu et al., 2015), the UK (Al‐Dujaili et al., 2022; Lynn et al., 2012; Tsang et al., 2012), Iran (Abedini et al., 2021; Akbarpour et al., 2021; Babaeian et al., 2013; Barati Boldaji et al., 2020; Esmaeilinezhad et al., 2019; Faghihimani et al., 2016; Goodarzi et al., 2021; Hosseini et al., 2016; Irani et al., 2020; Mirmiran et al., 2010; Nemati et al., 2022; Rouzbehan et al., 2021; Sohrab et al., 2014, 2019; Yarmohammadi & Mahjoub, 2017; Zarezadeh et al., 2019), Mexico (González‐Ortiz et al., 2011), South Korea (Park et al., 2014), Scotland (Stockton et al., 2017), Serbia (Kojadinovic et al., 2017, 2021), Greece (Manthou et al., 2017), and Bosnia and Herzegovina (Grabež et al., 2020). Seven studies were performed on females (Abedini et al., 2021; Akbarpour et al., 2021; Esmaeilinezhad et al., 2019; Kojadinovic et al., 2017; Park et al., 2014; Rouzbehan et al., 2021; Yarmohammadi & Mahjoub, 2017), two studies on males (Irani et al., 2020; Nemati et al., 2022), and the others were carried out on both genders (Al‐Dujaili et al., 2022; Babaeian et al., 2013; Barati Boldaji et al., 2020; Faghihimani et al., 2016; González‐Ortiz et al., 2011; Goodarzi et al., 2021; Grabež et al., 2020; Hosseini et al., 2016; Kojadinovic et al., 2021; Lynn et al., 2012; Manthou et al., 2017; Mirmiran et al., 2010; Sohrab et al., 2014, 2019; Stockton et al., 2017; Sumner et al., 2005; Tsang et al., 2012; Wu et al., 2015; Zarezadeh et al., 2019). The characteristics of the included studies are demonstrated in Table 1.

**TABLE 1:** Characteristic of included studies in meta‐analysis.

Studies	Country	Study design	Participant	Sex	Sample size		Trial duration (week)	Means age		Means BMI		Intervention		
					IG	CG		IG	CG	IG	CG	Type	Dose	Control group
Sumner et al. (2005)	USA	Parallel, R, DB, P	Coronary heart disease	Both	45	26	12	69	69	28	29	Juice	240 mL	Placebo
Mirmiran et al. (2010)	Iran	Parallel, R, DB, P	Hyperlipidemic	Both	45	23	4	51	55	27.2	28.3	Seed oil	400 mg	Placebo
González‐Ortiz et al. (2011)	Mexico	Parallel, R, DB, P	obese	Both	20	10	4	36.3 ± 8.3	38.3 ± 10.4	35.2 ± 3.1	33.8 ± 4.1	Juice	120 mL	Placebo
Tsang et al. (2012)	UK	Cross‐over, R, DB	at high CVD risk	Both	28	28	4	50.4	50.4	26.77	26.77	Juice	500 mL	Placebo
Lynn et al. (2012)	UK	Parallel, R, P	Healthy Young and Middle‐aged Men and Women	Both	48	24	4	39 ± 6.07	36.1 ± 4.50	24.99 ± 6.1	24.99 ± 5.19	Juice	330 mL	Placebo beverage
Babaeian et al. (2013)	Iran	Parallel, R, Open	T2DM	Both	45	23	8	46.5	47.5	28.56	28.48	Juice	240 mL	Water
Sohrab et al. (2014)	Iran	Parallel, R, DB, P	T2DM	Both	44	22	12	55 ± 6.7	56.9 ± 6.8	29.4 ± 3.9	28.6 ± 4.2	Juice	250 mL	Placebo
Park et al. (2014)	South Korea	Parallel, R, DB, P	overweight	Female	77	38	8	41 ± 2	42 ± 2	28.9 ± 0.3	28 ± 0.4	Juice	200 mL	Placebo
Wu et al. (2015)	USA	Parallel, R, DB, P	Hemodialysis	Both	27	13	24	52.6	55.9	32.3	31.1	Extract	1000 mg	Placebo
Hosseini et al. (2016)	Iran	Parallel, R, DB, P	overweight and obese individuals	Both	42	21	4	NR	NR	32.5 ± 4.1	31.1 ± 4.9	Extract	1000 mg	None
Faghihimani et al. (2016)	Iran	Parallel, R, DB, P	T2DM	Both	74	37	8	52 ± 6.8	48 ± 85	27 ± 2.4	26 ± 2.7	Seed oil	2000 mg	None
Yarmohammadi and Mahjoub (2017)	Iran	Parallel, R	T2DM	Female	16	9	6	56.5	49.5	27.09	26.77	Extract	150 mg	Water
Stockton et al. (2017)	Scotland	Parallel, R, DB, P	Healthy	Both	54	28	8	30.14 ± 10.95	34.11 ± 11.28	24.76 ± 3.83	23.57 ± 3.44	Extract	538.37 mg	Placebo/maltodextrin
Kojadinovic et al. (2017)	Serbia	Parallel, R	Metabolic Syndrome	Female	23	12	6	40–60	40–60	31.98	27.83	Juice	300 mL	Water
Manthou et al. (2017)	Greece	Parallel, R	Healthy	Both	10	5	2	31.8	31.8	NR	NR	Juice	500 mL	No juice
Zarezadeh et al. (2019)	Iran	Parallel, R, DB	Overweight and obese individuals	Both	42	21	4	30–60	30–60	32.5 ± 4.1	31.1 ± 4.9	Extract	1000 mg	Cellulose
Esmaeilinezhad et al. (2019)	Iran	Parallel, R, TB, P	PCOS	Female	46	23	8	29.3	30.6	26.25 ± 2.93	26.77 ± 1.70	Juice	300 mL	Placebo beverage
Sohrab et al. (2019)	Iran	Parallel, R, SB	T2DM	Both	60	30	6	54.6 ± 8.4	55.3 ± 8.5	27.2 ± 3.4	26.5 ± 3.6	Juice	200 mL	None
Grabež et al. (2020)	Bosnia & Herzegovina	Parallel, R, DB, P	T2DM	Both	37	19	8	57.58 ± 6.10	57.06 ± 6.73	31.82 ± 4.94	32.51 ± 4.72	Extract	500 mg	Placebo
Irani et al. (2020)	Iran	Parallel, R	Inactive students	Male	20	10	8	22	21	NR	NR	Extract	100 mg	Placebo
Barati Boldaji et al. (2020)	Iran	Cross‐over, R	Hemodialysis	Both	41	41	8	47.8	47.8	23.88	23.88	Juice	100 mL	Usual care
Goodarzi et al. (2021)	Iran	Parallel, R, DB, P	NAFLD	Both	44	22	12	47.41 ± 9.58	44.91 ± 9.41	30.76 ± 2.8	31.9 ± 3.01	Extract	225 mg	Placebo
Kojadinovic et al. (2021)	Serbia	Parallel, R	Overweight with Dyslipidemia	Both	24	12	2	54.42	52.38	29.02	27.85	Juice	300 mL	No juice
Abedini et al. (2021)	Iran	Parallel, R, Open	PCOS	Female	42	21	8	24.76	25.57	29.65	29	Juice	45 mL	Routine recommendations
Akbarpour et al. (2021)	Iran	Parallel, R	Breast cancer	Female	20	10	8	42.19	40.25	NR	NR	Juice	100 mL	Water
Rouzbehan et al. (2021)	Iran	Parallel, R, SB	Breast Cancer	Female	20	10	8	42.19	40.25	23.77	22.18	Juice	100 mL	Water
Al‐Dujaili et al. (2022)	UK	Parallel, R, SB	Healthy	Both	24	12	4	23.5	24	23.6	24.7	Extract	1000 mg	Maltodextrin
Nemati et al. (2022)	Iran	Parallel, SB, R, P	T2DM	Male	19	9	8	41.33 ± 1.50	41.81 ± 1.88	32.69 ± 1.30	33.34 ± 1.51	Juice	240 mL	Water
Nemati et al. (2022)	Iran	Parallel, SB, R, P	T2DM	Male	19	9	8	43.22 ± 2.48	42.60 ± 1.89	34.55 ± 3.14	32.28 ± 1.78	Juice	240 mL	Aerobic training

Abbreviations: CG, control group; CO, controlled; CVD, cardiovascular disease; DB, double‐blinded; IG, intervention group; NAFLD, non‐alcoholic fatty liver disease; NR, not reported; P, placebo‐controlled; PCOS, polycystic ovary syndrome; R, randomized; SB, single‐blinded; T2DM, type 2 diabetes mellitus; TB, triple‐blinded.

### 3.3 Quality assessment

The general risk of bias was evaluated and indicated in 23 studies with a good risk of bias (Akbarpour et al., 2021; Al‐Dujaili et al., 2022; Barati Boldaji et al., 2020; Esmaeilinezhad et al., 2019; Faghihimani et al., 2016; González‐Ortiz et al., 2011; Goodarzi et al., 2021; Grabež et al., 2020; Hosseini et al., 2016; Irani et al., 2020; Kojadinovic et al., 2021; Lynn et al., 2012; Manthou et al., 2017; Mirmiran et al., 2010; Park et al., 2014; Sohrab et al., 2014; Sohrab et al., 2019; Stockton et al., 2017; Sumner et al., 2005; Tsang et al., 2012; Wu et al., 2015; Yarmohammadi & Mahjoub, 2017; Zarezadeh et al., 2019), four trials with a fair risk of bias (Abedini et al., 2021; Babaeian et al., 2013; Kojadinovic et al., 2017; Rouzbehan et al., 2021), and a study showed a bad risk of bias (Nemati et al., 2022; Table 2).

**TABLE 2:** Risk of bias assessment.

Study	Random sequence generation	Allocation concealment	Selective reporting	Other sources of bias	Blinding (participants and personnel)	Blinding (outcome assessment)	Incomplete outcome data	General risk of bias
Sumner et al. (2005)	L	L	L	L	L	L	L	Good
Mirmiran et al. (2010)	L	L	L	L	L	L	L	Good
González‐Ortiz et al. (2011)	L	U	H	U	L	L	L	Good
Tsang et al. (2012)	U	L	L	L	L	L	L	Good
Lynn et al. (2012)	L	U	H	U	U	L	L	Good
Babaeian et al. (2013)	U	U	L	U	H	H	L	Fair
Sohrab et al. (2014)	L	L	L	L	L	U	L	Good
Park et al. (2014)	L	L	L	L	L	L	L	Good
Wu et al. (2015)	U	U	L	U	L	U	L	Good
Hosseini et al. (2016)	L	L	L	L	L	L	L	Good
Faghihimani et al. (2016)	L	L	L	L	L	L	L	Good
Yarmohammadi and Mahjoub (2017)	L	U	L	U	H	H	L	Good
Stockton et al. (2017)	L	L	H	L	L	U	L	Good
Kojadinovic et al. (2017)	U	U	L	H	H	U	L	Fair
Manthou et al. (2017)	U	U	L	L	U	U	L	Good
Zarezadeh et al. (2019)	L	U	L	L	L	U	L	Good
Esmaeilinezhad et al. (2019)	L	L	H	L	L	L	L	Good
Sohrab et al. (2019)	L	L	L	U	L	U	L	Good
Grabež et al. (2020)	L	U	L	L	L	L	L	Good
Irani et al. (2020)	L	U	L	U	U	U	L	Good
Barati Boldaji et al. (2020)	U	U	L	L	H	U	L	Good
Goodarzi et al. (2021)	L	L	L	L	L	L	L	Good
Kojadinovic et al. (2021)	L	U	L	L	U	U	L	Good
Abedini et al. (2021)	L	L	L	L	H	U	L	Fair
Akbarpour et al. (2021)	L	U	L	H	U	U	L	Good
Rouzbehan et al. (2021)	L	L	L	H	H	U	L	Fair
Al‐Dujaili et al. (2022)	L	L	L	L	H	U	L	Good
Nemati et al. (2022)	L	H	H	L	H	H	H	Bad

*Note*: General good < 2 high risk. General fair = 2 high risk. General bad > 2 high risk.

Abbreviations: H, high risk of bias; L, low risk of bias; U, unclear risk of bias.

### 3.4 Meta‐analysis

#### 3.4.1 Effect of pomegranate consumption on obesity indices in adults

##### Effect of pomegranate consumption on BMI

**FIGURE 2:**
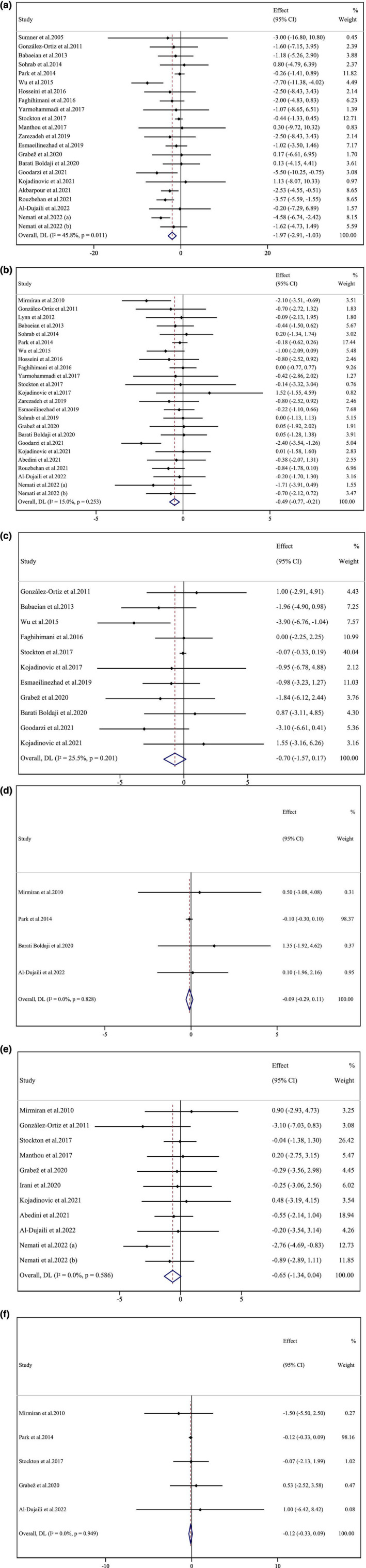
Forest plot detailing weighted mean difference and 95% confidence intervals (CIs) for the effect of pomegranate intake on (a) body weight (kg); (b) BMI (kg/m^2^); (c) WC (cm); (d) FM (kg); (e) BFP (%); and (f) FFM (kg).

A total of 24 studies investigated the impact of pomegranate intake on BMI. Pooled results from random effects model mentioned that BMI was lowered significantly following the pomegranate intake (WMD: −0.48 kg/m^2^ 95% CI: −0.76 to −0.20; *p* = .001; Figure 2b). An insignificant between‐studies heterogeneity was also reported (*I* ^2^ = 15.0%). Furthermore, subgroup analysis showed that BMI was lowered significantly following the long‐term intervention (≥8 weeks), high‐dose pomegranate extract intake (≥1000 mg/day), intervention by pomegranate extract as an intervention type, or pomegranate intake among individuals with metabolic disorders or obese participants (BMI > 30; Table 3).

**TABLE 3:** Subgroup analyses of pomegranate intake on body composition in adults.

	Number of effect sizes	WMD (95%CI)	*p*‐Value	Heterogeneity		
				P heterogeneity	*I* ^2^	P between subgroups
Pomegranate intake on serum body weight (kg)						
Overall effect	22	−1.97 (−2.91, −1.03)	**≤.001**	0.011	45.8%	
Trial duration (week)						
≥8	15	−2.10 (−3.21, −0.99)	**≤.001**	0.001	63.1%	0.600
<8	7	−1.35 (−3.94, 1.24)	.308	0.992	0.0%	
Intervention type						
Juice	13	−1.80 (−2.89, −0.71)	**.001**	0.086	37.2%	0.817
Seed oil	1	−2.00 (−4.83, 0.83)	.166	–	–	
Extract	8	−2.70 (−5.26, −0.14)	**.038**	0.010	61.9%	
Joice dose (mL/day)						
>200	8	−2.13 (−3.56, −0.70)	**.004**	0.356	9.5%	0.692
≤200	5	−1.69 (−3.35, −0.02)	**.047**	0.042	59.6%	
Extract dose (mg/day)						
≥1000	4	−3.99 (−7.57, −0.41)	**.029**	0.160	41.9%	0.225
<1000	4	−1.34 (−3.70, 1.00)	.262	0.234	29.8%	
Baseline BMI (kg/m^2^)						
Normal (18.5–24.9)	4	−1.36 (−3.47, 0.74)	.204	0.046	62.6%	.001
Overweight (25–29.9)	8	−0.57 (−1.50, 0.34)	.220	0.962	0.0%	
Obese (>30)	7	−4.38 (−6.09, −2.67)	**≤.001**	0.337	12.1%	
Sex						
Both	15	−1.78 (−3.15, −0.42)	**.010**	0.133	29.7%	0.600
Female	5	−1.71 (−3.22, −0.19)	**.027**	0.050	57.9%	
Male	2	−3.31 (−6.18, −0.45)	**.023**	0.126	57.3%	
Metabolic disorder						
No	8	−1.92 (−3.43, −0.40)	**.013**	0.001	71.6%	0.668
Yes	14	−2.32 (−3.38, −1.26)	**≤.001**	0.698	0.0%	
Pomegranate intake on serum BMI (kg/m^2^)						
Overall effect	24	−0.48 (−0.76, −0.20)	**.001**	0.253	15.0%	
Trial duration (week)						
≥8	14	−0.46 (−0.81, −0.11)	**.009**	0.184	25.1%	0.921
<8	10	−0.50 (−1.03, 0.03)	.064	0.501	0.0%	
Intervention type						
Juice	14	−0.24 (−0.53, 0.04)	.095	0.985	0.0%	.101
Seed oil	2	−0.96 (−3.01, 1.08)	.357	0.011	84.7%	
Extract	8	−0.97 (−1.60, −0.34)	**.002**	0.289	17.8%	
Joice dose (mL/day)						
>200	8	−0.20 (−0.71, 0.30)	.426	0.945	0.0%	0.861
≤200	6	−0.26 (−0.61, 0.08)	.140	0.818	0.0%	
Extract dose (mg/day)						
≥1000	4	−0.75 (−1.46, −0.03)	**.039**	0.867	0.0%	0.751
<1000	4	−1.01 (−2.47, 0.44)	.174	0.102	51.7%	
Baseline BMI (kg/m^2^)						
Normal (18.5–24.9)	5	−0.42 (−1.05, 0.21)	.191	0.832	0.0%	0.172
Overweight (25–29.9)	10	−0.23 (−0.53, 0.05)	.111	0.547	0.0%	
Obese (>30)	8	−0.98 (−1.72, −0.25)	**.008**	0.219	26.2%	
Sex						
Both	16	−0.56 (−0.98, −0.14)	**.008**	0.124	29.9%	0.518
Female	6	−0.26 (−0.61, 0.07)	.130	0.710	0.0%	
Male	2	−0.70 (−2.12, 0.71)	.330	0.925	0.0%	
Metabolic disorder						
No	7	−0.33 (−0.67, −0.01)	.057	0.750	0.0%	0.460
Yes	17	−0.53 (−0.94, −0.12)	**.011**	0.149	26.7%	
Pomegranate intake on serum WC (cm)						
Overall effect	11	−0.69 (−1.56, 0.17)	.116	0.201	25.5%	
Trial duration (week)						
≥8	8	−1.02 (−2.10, 0.06)	.065	0.084	44.1%	0.224
<8	3	0.76 (−1.90, 3.44)	.574	0.797	0.0%	
Intervention type						
Extract	4	−1.92 (−4.26, 0.41)	.106	0.017	70.7%	0.471
Juice	6	−0.50 (−1.89, 0.88)	.480	0.716	0.0%	
Seed oil	1	0.00 (−2.25, 2.25)	1.000	–	–	
Joice dose (mL/day)						
>200	4	−0.97 (−2.58, 0.62)	.233	0.674	0.0%	0.244
≤200	2	0.93 (−1.85, 3.72)	.510	0.964	0.0%	
Extract dose (mg/day)						
≥1000	1	−3.90 (−6.76, −1.04)	**.008**	–	–	0.092
<1000	3	−0.95 (−2.85, 0.94)	.327	0.175	42.7%	
Baseline BMI (kg/m^2^)						
Normal (18.5–24.9)	2	−0.06 (−0.32, 0.19)	.619	0.644	0.0%	0.052
Overweight (25–29.9)	4	−0.63 (−1.97, 0.70)	.355	0.571	0.0%	
Obese (>30)	5	−2.19 (−3.97, −0.41)	**.016**	0.357	8.8%	
Sex						
Both	9	−0.77 (−1.86, 0.32)	.166	0.119	37.5%	0.864
Female	2	−0.97 (−3.07, 1.12)	.361	0.992	0.0%	
Metabolic disorder						
No	3	−1.01 (−3.57, 1.55)	.440	0.029	71.7%	0.908
Yes	8	−0.84 (−1.97, 0.28)	.141	0.690	0.0%	
Pomegranate intake on serum fat mass (kg)						
Overall effect	4	−0.09 (−0.29, 0.11)	.374	0.828	0.0%	
Pomegranate intake on serum BFP (%)						
Overall effect	11	−0.64 (−1.33, 0.04)	.066	0.586	0.0%	
Trial duration (week)						
≥8	6	−0.76 (−1.58, 0.05)	.067	0.358	9.1%	0.558
<8	5	−0.23 (−1.79, 1.32)	.764	0.626	0.0%	
Intervention type						
Extract	4	−0.11 (−1.19, 0.96)	.835	0.998	0.0%	0.300
Juice	6	−1.13 (−2.15, −0.10)	.030	0.328	13.6%	
Seed oil	1	0.90 (−2.92, 4.72)	.645	–	–	
Joice dose (mL/day)						
>200	4	−1.13 (−2.60, 0.33)	.129	0.236	29.4%	0.984
≤200	2	−1.16 (−3.30, 0.97)	.286	0.238	28.1%	
Extract dose (mg/day)						
≥1000	1	−0.20 (−3.54, 3.14)	.907	–	–	0.958
<1000	3	−0.10 (−1.24, 1.03)	.857	0.984	0.0%	
Baseline BMI (kg/m^2^)						
Normal (18.5–24.9)	2	−0.06 (−1.30, 1.18)	.922	0.931	0.0%	0.064
Overweight (25–29.9)	3	−0.22 (−1.58, 1.13)	.745	0.728	0.0%	
Obese (>30)	3	−2.26 (−3.80, −0.73)	**.004**	0.401	0.0%	
Sex						
Both	7	−0.13 (−1.11, 0.83)	.779	0.851	0.0%	0.330
Female	1	−0.55 (−2.13, 1.03)	.496	–	–	
Male	3	−1.48 (−2.96, −0.00)	**.049**	0.254	27.1%	
Metabolic disorder						
No	4	−0.05 (−1.11, 1.00)	.919	0.997	0.0%	0.160
Yes	7	−1.07 (−2.02, −0.12)	.026	0.388	5.1%	
Pomegranate intake on serum fat‐free mass (kg)						
Overall effect	5	−0.12 (−0.33, 0.08)	.254	0.949	0.0%	

*Note*: Bold indicates statistical significance value (*p* < .05).

Abbreviations: BFP, body fat percentage; BMI, body mass index; CI, confidence interval; WC, waist circumference; WMD, weighted mean differences.

##### Effect of pomegranate consumption on body weight

The effects of pomegranate intake on body weight were assessed in 22 studies. Combined results from the random‐effects model indicated a significant reduction in body weight following the pomegranate intake (WMD: −1.97 kg 95% CI: −2.91 to −1.03; *p* ≤ .001; Figure 2a). A moderate heterogeneity was observed among studies (for body weight *I* ^2^ = 45.8%). This heterogeneity of 45.8% in body weight could suggest variations in study designs, duration, populations, or interventions that might influence the overall effect. The outcomes of subgroup analysis mentioned no significant decrease in body weight following the short‐term pomegranate intake (<8 weeks), low dose pomegranate extract intake (<1000 mg/day), or intervention among normal BMI or overweight participants. Additionally, using pomegranate seed oil or pomegranate extract as intervention types resulted in no significant reduction of body weight (Table 3).

##### Effect of pomegranate consumption supplementation on WC

According to the outcomes from 11 studies, pomegranate intake had no significant effect on WC (WMD: −0.69 cm; 95% CI: −1.56 to 0.17; *p* = .116; Figure 2c). Also, an insignificant degree of heterogeneity among studies was found (*I* ^2^ = 25.5%). In addition, assessing the outcomes of subgroup analysis showed that high‐dose pomegranate extract intake (≥1000 mg/day), or pomegranate intake among obese participants (BMI > 30), lowered WC (Table 3).

##### Effect of pomegranate consumption on FM

The effects of pomegranate consumption on fat mass were reported in four studies. Combined results from the random‐effects model revealed that pomegranate consumption failed to reduce the fat mass significantly (WMD: −0.09 kg; 95% CI: −0.29 to 0.11; *p* = .374; Figure 2d). In addition, there was no degree of between‐studies heterogeneity (*I* ^2^ = 0.0%; Table 3).

##### Effect of pomegranate consumption on BFP

Analyzing 11 overall effect sizes indicated that pomegranate intake failed to alter BFP significantly (WMD: −0.64%; 95% CI: −1.33 to 0.04; *p* = .066; Figure 2e). Also, no between‐studies heterogeneity was observed (*I* ^2^ = 0.0%). The results of the subgroup analysis indicated a significantly diminishing effect of pomegranate intake on BFP in obese (BMI > 30) or male participants, or in individuals with metabolic disorders. Moreover, with pomegranate juice as an intervention type, BFP was reduced significantly (Table 3).

##### Effect of pomegranate consumption on FFM

Pooled data from five studies indicated no significant change in FFM following the pomegranate intake (WMD: −0.12 kg; 95% CI: −0.33 to 0.08; *p* = .254; Figure 2f). Plus, no between‐studies heterogeneity was found (*I* ^2^ = 0.00%; Table 3).

#### 3.4.2 Sensitivity analysis

The exclusion of each trial from the review was performed in order to ascertain their impact on the overall effect size. As a result, by removing the study Stockton et al. (2017) (WMD: −0.86, CI95%: −1.66, −0.05), results mentioned a significant alteration of the overall effect size.

#### 3.4.3 Publication bias

**FIGURE 3:**
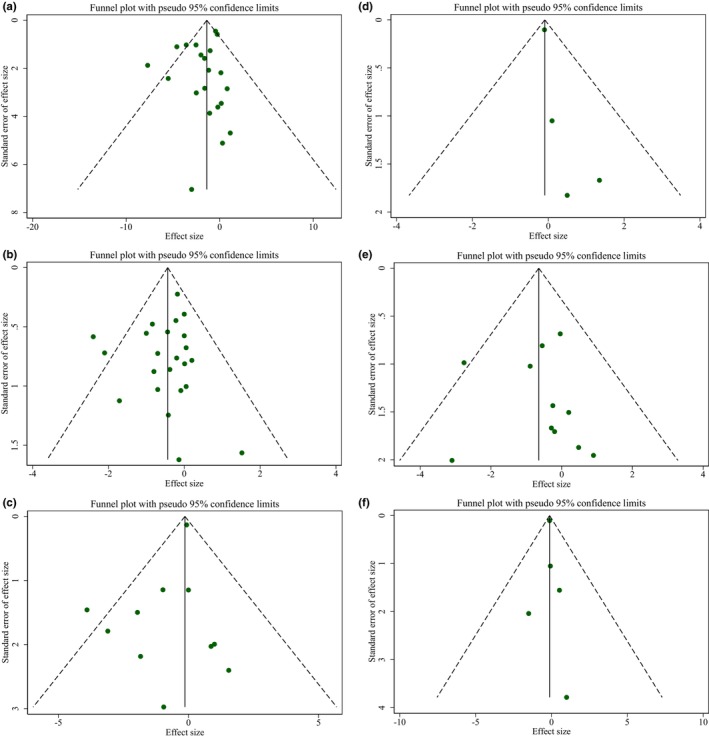
Funnel plots for the effect of pomegranate intake on (a) body weight (kg); (b) BMI (kg/m^2^); (c) WC (cm); (d) FM (kg); (e) BFP (%); and (f) FFM (kg).

The assessment of the results of Egger's regression test and the inspection of the funnel plots revealed no significant publication bias in studies evaluating the influence of pomegranate intake on body weight, BMI, WC, FM, BFP, and FFM. Additionally, Begg's examination demonstrated no significant publication bias (Figure 3).

#### 3.4.4 GRADE analysis

The results of the GRADE analysis mentioned a very high quality of the evidence in studies estimating the influence of pomegranate intake on BMI. Moreover, the evidence quality in studies evaluating the impact of pomegranate on body weight, WC, FM, BFP, and FFM. is regarded as high (Table 4).

**TABLE 4:** GRADE profile of pomegranate intake for body composition in adults.

Outcomes	Risk of bias	Inconsistency	Indirectness	Imprecision	Publication bias	Quality of evidence
Body weight	No serious limitation	Serious limitation^a^	No serious limitation	No serious limitation	No serious limitation	⊕ ⊕ ⊕◯ High
BMI	No serious limitation	No serious limitation	No serious limitation	No serious limitation	No serious limitation	⊕ ⊕ ⊕⊕ Very High
WC	No serious limitation	No serious limitation	No serious limitation	Serious limitation^b^	No serious limitation	⊕ ⊕ ⊕◯ High
FM	No serious limitation	No serious limitation	No serious limitation	Serious limitation^b^	No serious limitation	⊕ ⊕ ⊕◯ High
BFP	No serious limitation	No serious limitation	No serious limitation	Serious limitation^b^	No serious limitation	⊕ ⊕ ⊕◯ High
FFM	No serious limitation	No serious limitation	No serious limitation	Serious limitation^b^	No serious limitation	⊕ ⊕ ⊕◯ High

^a^ There is high heterogeneity (*I* ^2^ > 40%).

^b^ There is no evidence of significant effects of pomegranate intake.

## 4 DISCUSSION

The present systematic review and meta‐analysis quantified the effects of pomegranate consumption on obesity indices in 28 randomized controlled trials with a total number of 1124 adult participants (568 cases, 556 controls). The pooled analysis showed a significant reduction in body weight and BMI; however, no significant effect was found on WC, FM, FFM, and BFP.

The increasing prevalence of obesity in the world is a risk factor for non‐communicable diseases and a serious warning for public health (Blüher, 2019). A growing number of studies are investigating the impact of plants and herbs on obesity due to their safety and minimum adverse effects (Pothuraju et al., 2014). Results on the effect of pomegranate on anthropometric measures were inconsistent. We observed that pomegranate supplementation can significantly reduce body weight and BMI. In line with our results, several in vivo and in vitro studies reported a significant effect of pomegranate on lowering body weight. Supplementation with pomegranate seed oil resulted in the reduction of body weight, weight gain percentage, and leptin and an increase of adiponectin levels among a group of mice fed with a high‐fat diet for 14 weeks (McFarlin et al., 2008). An in vivo study showed that a dietary fatty acid derived from pomegranate seed oil suppressed adipogenesis by inhibiting the differentiation of human adipose‐derived mesenchymal stem cells into adipocytes (Trichur Khabeer et al., 2019). In contrast to our findings, a clinical study conducted by Heber et al. (2007) revealed that supplementation with pomegranate ellagitannin‐enriched polyphenol extract increased body weight and BMI. This result might be explained by the concurrence of the study with late fall holidays in the U.S. (Heber et al., 2007). Another clinical study reported that daily supplementation with pomegranate polyphenols for 4 weeks had no significant effect on body weight (Basu et al., 2013). Also, a quasi‐experimental study among type 2 diabetic patients revealed that consumption of concentrated pomegranate juice did not affect body weight, BMI, and WC (Shishehbor et al., 2016). Different findings might be explained by differences in study design, the absence of the required control group (Laurindo et al., 2022), dosage and duration of intervention, and participants' health status. Our subgroup analysis showed significant weight reduction when pomegranate was administered for more than 8 weeks to obese subjects. The effect of pomegranate on WC in obese participants and those who received pomegranate extract >1000 mg per day was more significant. Also, BFP decreased more in males and obese subjects with metabolic disorders. Intervention type is another important factor that determines the effect of pomegranate on anthropometric measures. Juice and extract forms were more efficient in lowering body weight and BFP in our analysis. Some studies even suggest that pomegranate peel has a stronger potential than pomegranate juice and pulp (Fahmy & Farag, 2022).

Our findings update the results of a meta‐analysis conducted by Gheflati et al. (2019) on the effect of pomegranate on body composition. They failed to find a significant role for pomegranate consumption in decreasing body weight, BMI, WC, and BFP compared to control arms (Gheflati et al., 2019). The lack of significance may have been due to the number of studies included in the previous meta‐analysis. In the present study, we included 15 other relevant published trials, and thus, our results are more comprehensive.

**FIGURE 4:**
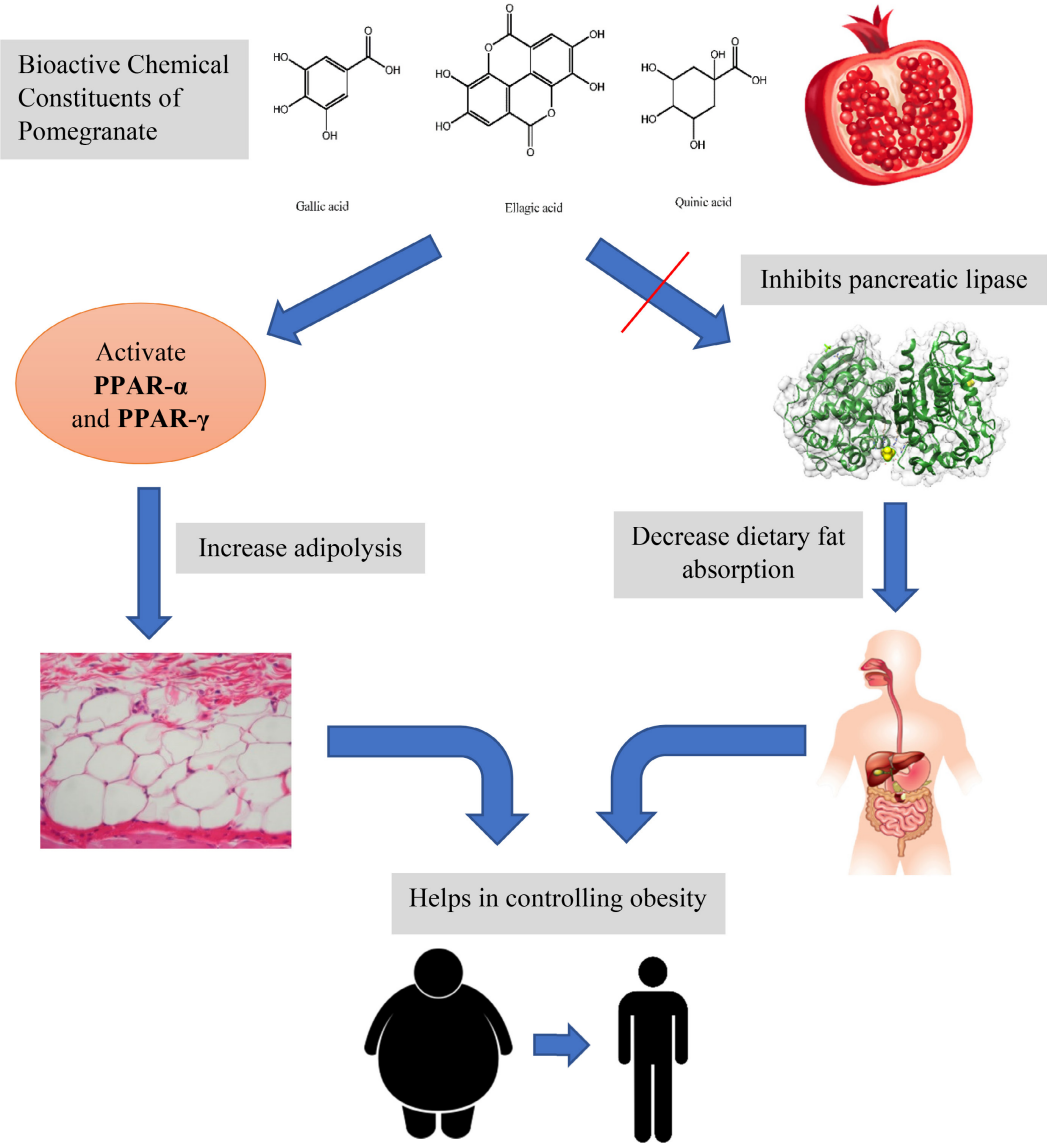
Anti‐obesity potential of pomegranate through activation of peroxisome proliferator‐activated receptors (PPAR‐γ and PPAR‐α) and inhibition of pancreatic lipase enzyme.

There are several mechanisms that could explain the effect of pomegranate on body composition. One mechanism is that pomegranate extract suppresses lipogenesis and Acetyl‐CoA production by reducing the expression of a major enzyme in lipogenesis, ATP citrate lyase (ACLY; Esmaeilinezhad et al., 2019). Furthermore, it is reported that pomegranate extract can increase energy consumption and thermogenesis in brown adipose tissue because it can act like peroxisome proliferator‐activated receptor α (PPAR‐α) agonist (Goodarzi et al., 2021; Rachid et al., 2015). PPAR‐α activation induces fatty acid oxidation in the liver and muscles and contributes to the improvement of lipid profile and reduction of adiposity (Evans et al., 2004). Another mechanism is that ellagic acid and tannic acid of pomegranate have similar effects as orlistat, that is, they inhibit pancreatic lipase activity, reduce dietary fat absorption in the blood, and increase fecal fat excretion (Lei et al., 2007; Figure 4). Pomegranate extract can also decrease appetite and calorie intake by decreasing leptin and elevating adiponectin levels (Al‐Muammar & Khan, 2012). Some animal studies also indicated that pomegranate polyphenols can have a favorable influence on obesity by modulating gut microbiota (Song, Shen, Chu, & Zheng, 2022; Song, Shen, Deng, et al., 2022). *Firmicutes* and *Bacteroidetes* are among the primary phyla in the gut microbiota. A higher ratio of *Firmicutes* to *Bacteroidetes* can lead to metabolic abnormalities such as obesity and diabetes (Singer‐Englar et al., 2019). It is suggested that pomegranate fruit pulp can reduce *Firmicutes* and increase *Bacteroidetes* proportion and thus, plays an anti‐obesity role (Song, Shen, Chu, & Zheng, 2022). It is worth mentioning that pomegranate components and antioxidants are able to modify symptoms of obesity since obesity is a chronic inflammatory state (Al‐Muammar & Khan, 2012).

The current study is an updated systematic review and meta‐analysis with a large number of eligible publications included. The results of Egger's and Begg's tests showed no evidence of publication bias. However, there are some limitations that should be acknowledged. First, the low number of studies on the effect of pomegranate on FM and FFM does not let us reach a firm conclusion. Second, it is important to keep in mind that the majority of the included studies did not evaluate the effect of pomegranate on body composition as the primary outcome (Gheflati et al., 2019). Third, despite the fact that we did subgroup analysis, the included studies were conducted among subjects with various health conditions, and this should be considered when interpreting the results. Therefore, more high‐quality studies with a focus on body composition are required.

In conclusion, we observed a significant reduction in body weight and BMI following pomegranate supplementation. However, we did not find any significant effect of pomegranate on WC, FM, FFM, and BFP. Statistically, pomegranate consumption reduced BMI and body weight significantly, however, this decrease was not clinically meaningful.

## AUTHOR CONTRIBUTIONS

**Hossein Bahari:** Conceptualization (lead); data curation (equal). **Sanaz Pourreza:** Validation (equal); writing – original draft (equal). **Kian Goudarzi:** Formal analysis (equal). **Seyedeh Nooshan Mirmohammadali:** Investigation (equal); writing – original draft (equal). **Omid Asbaghi:** Investigation (equal); methodology (equal); project administration (equal). **Kosar Sadat Hosseini Kolbadi:** Data curation (equal); visualization (equal). **Moslem Naderian:** Writing – review and editing (equal). **Ali Hosseini:** Writing – review and editing (equal).

## CONFLICT OF INTEREST STATEMENT

The authors declare that they have no competing interests.

## ETHICS STATEMENT

Not applicable.

## CONSENT FOR PUBLICATION

Not applicable.

## Data Availability

Data will be available on request from the authors.
